# Supplementary material for: Global hypo-methylation in a proportion of glioblastoma enriched for an astrocytic signature is associated with increased invasion and altered immune landscape
Source: eLife. 2022 Nov 22;11:e77335. doi: 10.7554/eLife.77335 (PMC9681209; doi:10.7554/eLife.77335)
Supplement: Figure 2—source data 1. [file elife-77335-fig2-data1.zip › Figure_2_source_data_1/Figure_2B/homerResults/motif40.similar.html]

motif40

## Information for motif40

A
T
C
G
G
C
A
T
T
G
C
A
G
C
T
A
T
C
A
G
A
T
G
C
T
A
G
C
C
G
T
A
T
C
A
G
G
T
A
C
T
G
A
C
T
C
G
A
A
G
T
C
G
A
C
T
G
T
C
A
A
G
T
C
T
G
C
A
T
G
A
C
G
T
A
C
C
G
T
A
T
A
C
G
C
A
G
T
T
G
C
A
C
G
T
A
C
T
A
G
T
G
A
C
G
A
T
C
T
C
G
A
A
T
C
G
T
A
G
C
G
A
T
C
T
C
G
A
A
T
G
C
G
A
C
T
C
T
G
A
  
Reverse Opposite:  

G
A
C
T
C
T
G
A
T
A
C
G
A
G
C
T
C
T
A
G
A
T
C
G
T
A
G
C
A
G
C
T
C
T
A
G
A
C
T
G
G
A
T
C
G
C
A
T
A
C
G
T
G
T
C
A
A
T
G
C
G
C
A
T
C
A
T
G
A
C
T
G
A
C
G
T
T
C
A
G
C
A
G
T
C
T
G
A
T
C
A
G
A
G
C
T
A
C
T
G
C
A
T
G
A
G
T
C
G
C
A
T
A
T
C
G
T
A
C
G
A
G
T
C
C
G
A
T
A
C
G
T
C
G
T
A
T
A
G
C
  

|  |  |
| --- | --- |
| p-value: | 1e-38 |
| log p-value: | -8.929e+01 |
| Information Content per bp: | 1.755 |
| Number of Target Sequences with motif | 84.0 |
| Percentage of Target Sequences with motif | 3.63% |
| Number of Background Sequences with motif | 2.0 |
| Percentage of Background Sequences with motif | 0.58% |
| Average Position of motif in Targets | 96.8 +/- 46.5bp |
| Average Position of motif in Background | 159.0 +/- 0.0bp |
| Strand Bias (log2 ratio + to - strand density) | 0.4 |
| Multiplicity (# of sites on avg that occur together) | 1.10 |
| Motif File: | file (matrix) reverse opposite |

### Similar de novo motifs found

|  |  |  |  |  |  |  |  |
| --- | --- | --- | --- | --- | --- | --- | --- |
| Rank | Match Score | Redundant Motif | P-value | log P-value | % of Targets | % of Background | Motif file |
| 1 | 0.840 | A G T C C G A T G C T A A G T C C T G A A G T C T A G C C G T A A C T G A C G T T G C A G C T A T C A G G T A C A T G C C G T A A C T G G T A C T G A C T C G A G T A C A C G T T G C A G A T C G T C A G T A C G T A C G C T A T A C G A G C T G T C A G C T A C T A G G A T C G T A C G C T A T A C G T G A C G A T C C T G A | 1e-12 | -28.246954 | 3.50% | 1.66% | motif file (matrix) |
| 2 | 0.911 | T C G A C G T A C T A G A G T C G T A C C G T A T A C G A G T C G T A C T C G A A G T C A G C T G C T A G A T C G C T A A T G C A G T C C T G A C A T G C A G T T C G A G C T A T C A G G T A C T A G C C T G A T C A G G A T C G A T C C T G A | 1e-11 | -25.479971 | 3.38% | 1.63% | motif file (matrix) |
